# Harnessing the Therapeutic Potential of Mesenchymal Stem Cells in Cancer Treatment

**DOI:** 10.34172/apb.2024.052

**Published:** 2024-06-22

**Authors:** Parisa Kangari, Reza Salahlou, Somayeh Vandghanooni

**Affiliations:** ^1^Department of Applied Cell Sciences, Faculty of Advanced Medical Sciences, Tabriz University of Medical Sciences, Tabriz, Iran.; ^2^Hematology and Oncology Research Center, Tabriz University of Medical Sciences, Tabriz, Iran.; ^3^Department of Medical Biotechnology, Faculty of Advanced Medical Sciences, Tabriz University of Medical Sciences, Tabriz, Iran.; ^4^Research Center for Pharmaceutical Nanotechnology, Biomedicine Institute, Tabriz University of Medical Sciences, Tabriz, Iran.

**Keywords:** Mesenchymal stem cells, Cancer therapy, Cell therapy, Exosomes, Immunomodulation, Chemotherapeutic agents

## Abstract

Cancer, as a complicated disease, is considered to be one of the major leading causes of death globally. Although various cancer therapeutic strategies have been established, however, some issues confine the efficacies of the treatments. In recent decades researchers for finding efficient therapeutic solutions have extensively focused on the abilities of stem cells in cancer inhibition. Mesenchymal stem cells (MSCs) are multipotent stromal cells that can the most widely extracted from various sources such as the bone marrow (BM), placenta, umbilical cord (UC), menses blood, Wharton’s jelly (WJ), adipose tissue and dental pulp (DP). These cells are capable of differentiating into the osteoblasts, chondrocytes, and adipocytes. Due to the unique characteristics of MSCs such as paracrine effects, immunomodulation, tumor-tropism, and migration, they are considered promising candidates for cancer therapeutics. Currently, MSCs are an excellent living carrier for delivery of therapeutic genes and chemical agents to target tumor sites. Also, exosomes, the most important extracellular vesicle released from MSCs, act as a strong cell-free tool for cancer therapeutics. MSCs can prevent cancer progression by inhibiting several signaling pathways, such as wnt/β-catenin and PI3K/AKT/mTOR. However, there are several challenges associated with the use of MSCs and their exosomes in the field of therapy that need to be considered. This review explores the significance of MSCs in cell-based therapy, focusing on their homing properties and immunomodulatory characteristics. It also examines the potential of using MSCs as carriers for delivery of anticancer agents and their role in modulating the signal transduction pathways of cancer cells.

## Introduction

**Figure 1:**
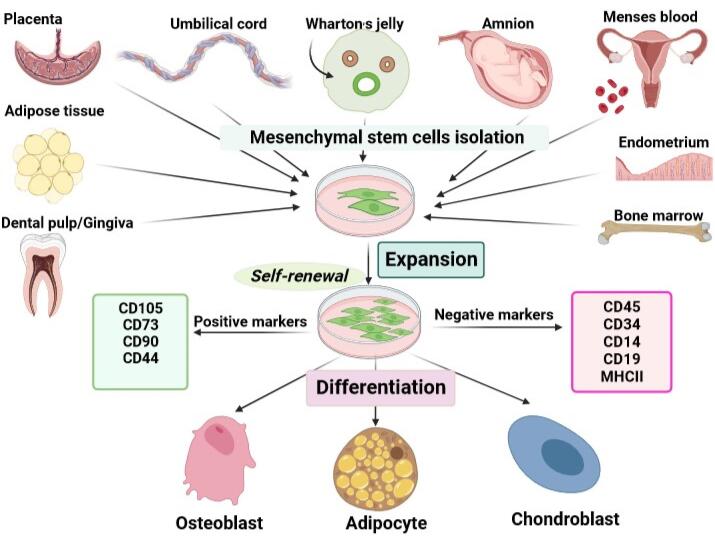
Mesenchymal stem cells sources and characterization. The web-based application BioRender was employed to design the figure.MSCs are isolated from various tissue sources such as dental pulp, adipose tissue, placenta, umbilical cord, Wharton’s jelly, menses blood, endometrium, and bone marrow. These plastic adherent cells are identified by their ability for trilineage differentiation, consistent expression of nonhematopoietic cell markers such as CD105, and CD73, and absence of hematopoietic cell markers such as CD34, CD 45, etc

 Currently, cancer is a major and widespread health concern, contributing significantly to global mortality and morbidity across various populations.^1^ According to the latest global cancer statistics in 2022, the most prevalent malignancies around the world include breast cancer in women (11.7%), lung cancer (11.4%), colorectal cancer (10.0%), prostate cancer (7.3%), and stomach cancer (5.6 %).^2^ The conventional cancer treatment methods include surgery, chemotherapy, immunotherapy, and radiation therapy that can be used alone or in combination. However, multiple deficiencies, such as significant adverse effects, off-target effects of therapeutic agents, and drug resistance restrict the efficacies of the therapeutic modalities.^3,4^ Recently, to dominate the barriers and disadvantages correlated with traditional treatments in cancer, cell therapy as one of the most prominent emerging medical treatments has been extensively considered.^5^ Mesenchymal stem cells (MSCs) are an appealing resource for cell-based therapy in a wide range of diseases including cancer due to their distinct characteristics.^6^ MSCs are fibroblast-like multipotent adult stem cells that can easily be derived, without ethical conflicts, from different tissues including bone marrow (BM), placenta, umbilical cord (UC), UC blood, menses blood, endometrium, Wharton’s jelly (WJ), adipose tissue, amnion, dental pulp (DP), etc.^7^ According to the definition of the International Society for Cell Therapy (ISCT), MSCs are characterized as plastic-adherent cells, with trilineage differentiation ability (osteoblasts, adipocytes, and chondroblasts), positive for CD105, CD73, and CD90, whereas negative for hematopoietic markers, e.g., CD45, CD34, CD14, CD19 and MHC class II (Figure 1).^8^ In addition, MSCs-based therapeutic approaches provide a promising platform for the treatment of incurable diseases including cancer due to their pleiotropic activities such as paracrine effects, immunomodulation, tumor-tropism, tumor-homing, and migration.^9^ Lazarus et al performed the first clinical trial of culture-expanded MSCs in patients with hematologic malignancies. They showed that expansion and following infusion of human bone marrow-derived stromal progenitor cells (BM-MSCs) in patients caused no intensive side effects.^10^ Because of the many benefits of MSCs, extensive preclinical (Table 1) and clinical studies have been conducted in a variety of cancer (Table 2). The present review has focused on the mechanisms of MSCs in inhibiting the progression of cancer and has explored the therapeutic approaches for treating cancer using MSCs.

**Table 1:** Preclinical studies of MSCs for the treatment of cancer

**Condition**	**Model**	**Treatment agent**	**Findings**
Breast cancer	Nude Mice	rBM-MSCs + PS-SiO 2 NPs	The suppression of tumor growth, resulting from injecting PS-loaded MSCs, is due to the naturally high affinity of MSCs toward tumors.^11^
Lung cancer	Nude Mice Rabbit Monkey	MSCs/NP/DTX	The lung targeting ability of MSC in different animal models is highly efficient for MSCs/NP/DTX systems in tumor inhibition rather than NP/DTX.^12^
Breast cancer	Mice	Mouse BM-MSCs-derived exosomes	Downregulation of VEGF expression in tumor cells, inhibition of angiogenesis, and tumor growth.^13^
Multiple myeloma	SCID Mice	pIL6-TRAIL-engineered hUC-MSC	Induction of apoptosis in MM cells.^14^
Kaposi's sarcoma	Nude mouse	hMSCs-CXCR4/Fluc2	Inhibition of Akt activity within KS cells and tumor-suppression.^15^
Hepatoma	SCID Mice	HMSCs	Inhibition of cancer cell phenotypes.^16^

rBM-MSCs: Rat bone marrow-derived mesenchymal stem cells, PS: Photo-sensitizer, SiO 2 NPs: Silica nanoparticles, MSCs: Mesenchymal stem cells, BM-MSCs: Bone marrow-derived mesenchymal stem cell, DTX: Docetaxel, NP: Nanoparticle, VEGF: vascular endothelial growth factor, SCID: Severe combined immunodeficiency, pIL6: Interleukin-6 promoter, TRAIL: transduced to express the tumor necrosis factor-related apoptosis-inducing ligand, hUC-MSCs: Human umbilical cord-derived mesenchymal stem cells, MM: Multiple myeloma, KS: Kaposi's sarcoma, CXCR4/Fluc2: CXC chemokine receptor 4/ firefly luciferase2, hMSCs: Human mesenchymal stem cells.

**Table 2:** Clinical studies of cancer treatment using MSCs

**Trial NCT number**	**Condition**	**Treatment agent**	**Phase**	**Start date**	**Status**	**Results**
NCT01129739	Myelodysplastic syndromes	hUC/PL-MSCs	II	2010	Unknown	No results available
NCT01092026	Hematological malignancies	UCB-HSCT with MSCs	I, II	2010	Completed	No results available
NCT01045382	Hematological malignancies	MSCs	II	2010	Terminated	No results available
NCT01844661	Solid tumors metastases	CELYVIR (BM- MSCs infected with ICOVIR5 (oncolytic virus)	I, II	2013	Completed	A reasonably safe and well-tolerated medication that may help patients with advanced malignancies experience a therapeutic response
NCT01983709	Prostate cancer	Allogeneic BM-MSC	I	2013	Terminated	No results available
NCT02270307	Hematological malignancies	MSCs and Cyclophosphamide	II, III	2014	Unknown	No results available
NCT02079324	Head and neck cancer	Genetically Modified Mesenchymal Stem Cells (GX-051)	I	2014	Unknown	No results available
NCT03106662	Hematological malignancies	MSCs	III	2014	Completed	No results available
NCT02513238	head and neck cancer	ASCs	II	2015	Completed	Primary results include changes in salivary flow rate, with secondary outcomes focusing on safety, quality of life, and gland evaluations using MRI and core-needle samples.
NCT02530047	Ovarian cancer	BM-MSCs-INFβ	I	2016	Completed	No results available
NCT02648386	Rectal cancer	HUC-MSCs	I, II	2016	Unknown	No results available
NCT03896568	Glioma	Allogeneic BM- MSCs loaded with the oncolytic adenovirus DNX-2401(BM-MSCs-DNX2401)	I	2019	Recruiting	Study is ongoing
NCT03298763	Adenocarcinoma of lung	MSC-TRAIL	I, II	2019	Recruiting	Study is ongoing
NCT04657315	Recurrent glioblastoma	MSC11FCD the suicide gene, cytosine deaminase	I, II	2020	Completed	No results available
NCT03608631	Pancreatic cancer	MSCs-derived Exosomes with KRAS G12D siRNA (iExosomes)	I	2021	Recruiting	Reduction of STAT3 levels, Inhibition of ECM deposition, and improving liver function in mice with liver fibrosis, presenting a promising anti-fibrotic therapeutic approach.
NCT03184935	Myelodysplastic syndromes	Allogeneic hUC-MSCs	I, II	2023	Suspended	Study is ongoing

MSCs: Mesenchymal stem cells, hUC/PL-MSCs: Human umbilical cord/placenta-derived MSCs, UCB-HSCT: Umbilical cord blood-hematopoietic stem cell transplantation, hUC-MSCs: Human umbilical cord-derived mesenchymal stem cells, BM-MSCs: Bone marrow-derived mesenchymal stem cells, ASCs: Adipose tissue-derived mesenchymal stem cells

## Mechanisms of MSCs function in cancer management

###  The tumor tropism and migration, homing properties of MSCs

**Figure 2:**
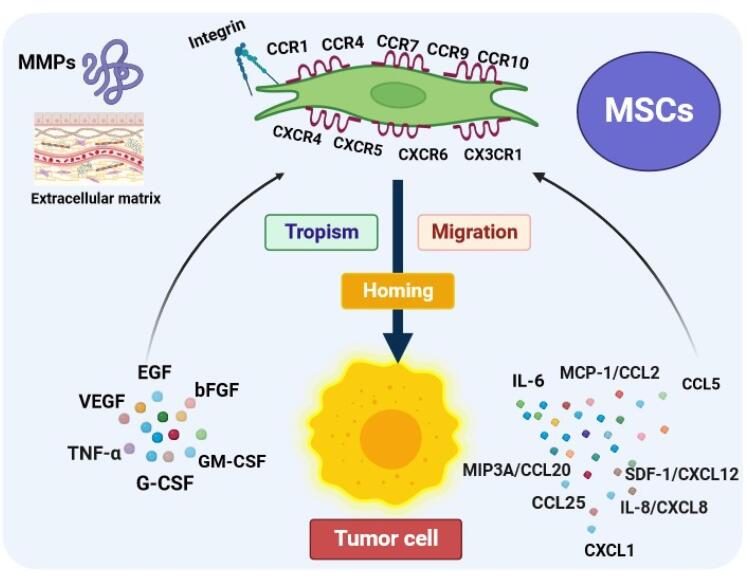
The mechanisms of mesenchymal stem cells tropism, migration, and homing. The web-based application BioRender was employed to design the figure.MSCs areattracted into the tumor microenvironment by interacting with multiple factors including chemokines, cytokines, and growth factors released by tumor cells. Moreover, the expression of some proteins such as integrin and matrix metalloproteinases (MMPs) in MSCs can favor tropism/migration and homing features of them. interleukin-6 (IL-6), monocyte chemotactic protein1(MCP-1/CCL2), Chemokine (C-C motif) ligand 25 (CCL25), macrophage inflammatory protein-3 (MIP3A/CCL20), C-X-C motif chemokine ligand 1 (CXCL1), interleukin-8 (IL-8/CXCL8), stromal-derived factor 1(SDF-1/CXCL12), vascular endothelial growth factor (VEGF), epidermal growth factor (EGF), basic fibroblast growth factor (bFGF), tumor necrosis factor (TNF-α), granulocyte colony-stimulating factor(G-CSF), granulocyte-macrophage colony-stimulating factor (GM-CSF)

 For juxtacrine effects (cell-to-cell or cell-to-ECM signaling) to occur, the migration and homing of MSCs to the damaged tissue are essential initial stages for the treatment of cancer in MSCs-based therapy. Numerous studies have demonstrated the natural affinity of MSCs for tumors, as well as their ability to migrate and home in on tumor tissues. This makes stem cells an exceptional therapeutic approach for targeting cancer cells. It has been found that the recruitment of MSCs towards tumor cells is thought to be due paracrine signaling loop between the chemoattractants from the tumor microenvironment (TME) and the corresponding receptors on MSCs.^17,18^ The inflammatory microenvironment of malignant cells due to the secretion of factors including growth factors, chemokines, and cytokines plays a substantial role in stimulating tumor tropism of MSCs.^19^ The most plentiful secreted chemokines in the TME are interleukin-6 (IL-6), monocyte chemotactic protein(MCP-1/CCL2), CCL15, macrophage inflammatory protein-3 (MIP3A/CCL20), CCL25, C-X-C motif chemokine ligand 1 (CXCL1), interleukin-8 (IL-8/CXCL8), stromal-derived factor 1(SDF-1/CXCL12) that contribute to the recruitment of MSCs derived from different sources toward cancer cells through interaction with their specific chemokine receptors at their surface.^20-22^ Further, several other trophic factors released from tumor cells and stroma such as vascular endothelial growth factor (VEGF), epidermal growth factor (EGF), basic fibroblast growth factor (bFGF), tumor necrosis factor (TNF-α), granulocyte colony-stimulating factor (G-CSF), and granulocyte-macrophage colony-stimulating factor (GM-CSF) induce MSCs attraction and homing in tumor sites.^23,24^ Furthermore, MSCs exhibit a remarkable tendency to migrate toward cancer through the expression of a large number of molecules including chemokines and their receptors, growth factors, toll-like receptors (TLRs), adhesion molecules, and growth factors.^25^ MSCs can express a variety range of functional chemokine receptors such as CCR1, CCR4, CCR7, CXCR4, CXCR6 which have been extensively related to tumors tropism.^26,27^ Based on a growing body of evidence, CXCR4 is one of the most important chemokine receptors in MSCs that plays a pivotal role in targeted homing of MSCs through interaction with stromal cell-derived factor 1 (SDF-1).^28^ In addition, the expression of different growth factor receptors on MSCs is involved in the migration and homing process. For example, c-met (HGF-R) is expressed on MSCs derived from cord blood, bone marrow, adipose tissue, cord blood, and skin and can induce cell migration and homing to the target site by binding to hepatocyte growth factor (HGF). To date, a multitude of studies have shown that the expression of PDGFR α and β, EGF-R, and IGF-R1 in BM-MSCs allows homing of these cells.^29-31^ MSCs recruitment into injured tissue is dependent on the expression of a variety of adhesion molecules including integrins. MSCs have been shown to express various integrin subunits such as α1, α2, α3, α4, α5, αv, β1, β3, and β4.^32^ Integrin heterodimer α4/β1, composed of CD49d and CD29, plays a crucial role in MSCs rolling and migration into targeted sites via binding to the vascular cell adhesion molecule 1 (VCAM-1) or CD106 expressed on endothelial cells.^33^ Matrix metalloproteinases (MMPs), zinc-dependent proteolytic enzymes, play a pronounced role in regulating the migratory activity of MSCs.^34^ Ho and colleagues reported for the first time that the expression of MMP-1 on BM-MSCs is most vital for the migration of these cells onto human glioma via the MMP1/PAR1 axis.^35^ MMP-1 can be considered as an IGFBP2 protease which may regulate MSCs tropism and migration to tumor through cleavage of IGF‐2/IGFBP 2 complex and extracellular release of free IGF‐2.^36^ Furthermore, it has been shown that blocking MMP-2 in tumor cells leads to the inhibition of human UC blood-derived MSCs attraction to tumor sites by preventing SDF1/CXCR4 signaling (Figure 2).^37^ While the recruitment of MSCs into the TME through growth factors and cytokines released by cancerous cells implicates tumor suppression, the interaction between MSCs and tumor cells may also contribute to the advancement of cancer.^38^ For instance, MSCs can differentiate into cancer-associated fibroblasts (CAFs) by cancer microenvironment-derived TGF-β, WNT, and IL-6/STAT3 signaling, increasing tumor cell heterogeneity and directly being involved in the progression of cancer.^39-41^ Accumulating evidence has indicated that MSCs, by upregulating EMT markers including vimentin, Twist, N-cadherin, and Snail and downregulating E-cadherin, could trigger cancer cell metastasis.^42,43^ It is also reported that MSCs have the ability to accelerate cancer progression by the secretion of multiple growth factors such as VEGF and bFGF and the prevention of apoptosis in tumor cells.^17^ In addition, MSCs were found to play a supportive role in tumor proliferation and promotion via the release of IL8 and the recruitment of leukocytes, such as macrophages and neutrophils.^44,45^ Consequently, the presence of MSCs in TME can have contradictory effects.^46^ Even though the precise mechanisms of MSCs migration to sites of tumor are not yet completely understood, the tumor-seeking behavior of MSCs has been utilized to develop more specific and efficient anticancer therapeutic strategies.

###  Immunomodulatory

**Figure 3:**
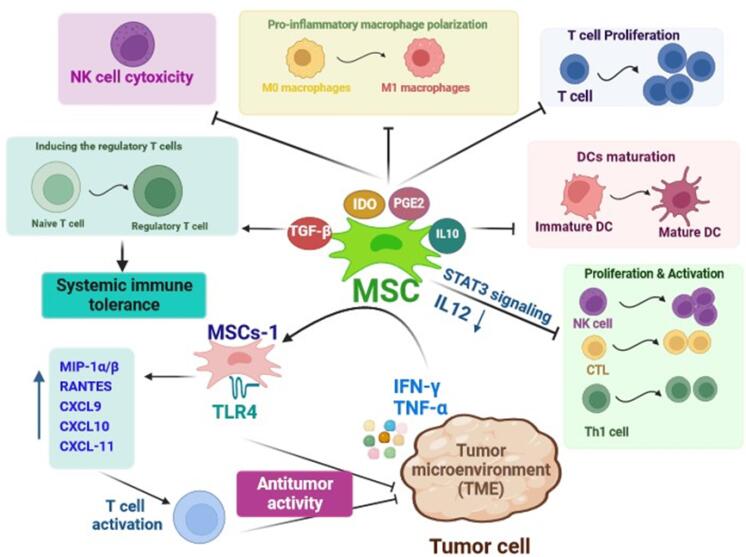
Schematic summarizing the immunomodulatory mechanisms of mesenchymal stem cells in cancer therapy. The web-based application BioRender was employed to design the figure. MSCs produce a variety of soluble factors that by interaction with immune cells activate or suppress their function and display immunomodulatory effects. macrophage Inflammatory Protein -1α/β(MIP-1α/β), regulated upon activation, normal T cell expressed and presumably secreted(RANTES), C-X-C Motif Chemokine Ligand 1(CXCL), toll-like receptor 4(TLR 4), interferon‐gamma (IFN‐γ), Tumor necrosis factor α (TNFα) indoleamine 2,3-dioxygenase(IDO), transforming growth factor-β (TGF-β), prostaglandin E2 (PGE2), Interleukin(IL), dendritic cell (DC), natural killer cells(NK cell), cytotoxic T lymphocytes (CTL), T helper cells (Th1 cell)

 MSCs mainly exert low immunogenicity and reveal a remarkable capacity to modulate immune responses.^47^ In a study by Bartholomew and colleagues, the immunomodulatory potential of baboon-derived MSCs was demonstrated through the inhibition of allogeneic peripheral blood lymphocyte proliferation and also the prevention of rejection in a baboon skin allograft model in vivo.^48^ It is well proven that MSCs elicit the immunomodulation functions through various mechanisms including direct interaction with immune cells and mediation of paracrine activity.^47^ The different kinds of innate immune cells such as dendritic cells (DCs), natural killer (NK) cells, monocytes/ macrophages, neutrophils, and adaptive immune cells such as T cells and B cells are suppressed or activated by MSCs (Figure 3).^49^ A significant focus has been placed on the paracrine effects of MSCs in this context as multiple factors derived from MSCs including cytokines and exosomes are related to their immunomodulatory capacity.^50^ For example, MSCs-derived indoleamine 2, 3-dioxygenase (IDO) and prostaglandin E2 (PGE2) inhibit the polarization of pro-inflammatory macrophage, proliferation of T cells, and cytotoxic activity of NK cells. The MSC-derived transforming growth factor-β (TGF-β) plays an indispensable role in maintaining systemic immune tolerance by promoting the induction of regulatory T cells (T reg).^51^ Additionally, MSCs can inhibit the maturation of DCs by releasing IL-10 and activating the signal transducer and activator of transcription (STAT) 3 signaling. This leads to reduced IL-12 production by DCs, which in turn prevents the proliferation and activation of NK cells, cytotoxic T cells (CTLs), and type 1 T helper (Th1) cells.^47^ The immunomodulatory effects of MSCs are primarily influenced by the solid tumor environment. This suggests that the immune regulatory responses induced by MSCs may vary or change depending on the surrounding microenvironment in which they are present.^52,53^ In addition to the ability of MSCs to modulate immune responses, they can regulate immunocompetence via stimulating immune cells to recruit into inflammatory conditions.^54^ MSCs can exhibit either pro-inflammatory or anti-inflammatory functions in an inflammatory environment. This is based on the levels or amounts of various factors they secrete or release into the surrounding environment.^55^ Accordingly, Waterman et al, suggested a new type of MSCs in which MSCs-1 express toll-like receptor 4 (TLR 4), and exert antitumor activity, while, due to the expression of TLR3, MSCs-2 inhibit immune cell activity and support tumor growth.^56^ The investigations have shown that MSCs adopt a pro-inflammatory phenotype or characteristics in the presence of low levels of the IFN-γ and TNF-α. In such environments, MSCs secret certain soluble factors such as MIP-1α/β, RANTES, CXCL9, CXCL10, and CXCL-11 to further activate T cells. Furthermore, in the absence of the IL-6, but in the presence of IFN-γ and IL-1, MSCs can activate M1 macrophages. These macrophages then further release high levels of IFN-γ and TNF-α within the damaged tissue environment.^57^ The findings of an in vivo study carried out by Ohlsson et al showed that co-administration of cancer cells and MSCs facilitated infiltration of monocytes and granulocytes in contrast to tumor cells or mesenchymal progenitor cells alone. This research demonstrated that MSCs are capable of preventing colon carcinoma growth.^58^ Further, it has been found that MSCs can cause cancer cell death through the production of unique immunomodulatory cytokines. For example, it was demonstrated that the overexpression of IL-12 in MSCs increased antitumor responses of T cells and repressed tumor growth.^59^ Due to the immunomodulatory trait of MSCs, they are recognized as an excellent focal point in anticancer therapeutic strategies.

## Current strategies in MSCs-based cancer therapy

 Exploitation of innate abilities of MSCs including tumor tropism, homing, and immunomodulatory endows new and innovative applications for inflammatory disorders including cancer. Recently, significant research attempts have concentrated on the use of stem cells as the vehicle for to targeted delivery of anti-cancer agents to tumor cells. Moreover, many studies have highlighted that MSCs-derived exosomes can serve as a potent cell-free tool for cancer treatment.^60^ Resistance against anti-cancer payloads is one of the most substantial barriers in the treatment of divergent types of solid tumors. Resistance develops due to the continuing utilization and elevated concentration of anti-cancer compounds, which augment the toxicity of anti-cancer drugs in noncancerous proliferating cells.^61^ Inadequate selectivity of a variety of anticancer therapeutic agents could be responsible for the problem.^62^ To overcome the mentioned problems, MSCs have been considered an appropriate vehicle for the targeted delivery of chemotherapeutic drugs, suicide genes, oncolytic viruses (OVs), cytokines, and growth factors to tumor cells, because of their intrinsic ability in tumor tropism and deep migration into the TME.^63^

###  MSCs as a delivery system for chemotherapeutic agents

**Figure 4:**
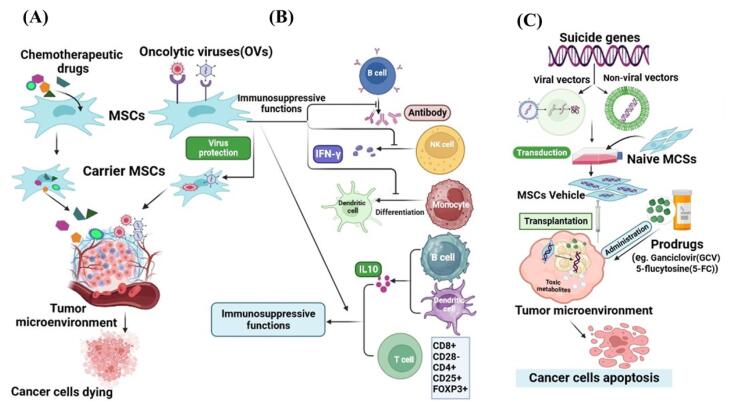
Mesenchymal stem cells as an appropriate carrier for drug agents, viruses, and gene delivery to tumor microenvironment. The web-based application BioRender was employed to design the figure. MSCs due to inherent tropism and high migration potential to cancer cells are convincing vehicles to load **(A)** chemotherapeutic drugs, **(B)** oncolytic viruses, and **(C)** suicide genes. MSCs with immunosuppressive ability protect from oncolytic viruses against immune cells **(B)**. Various suicide genes have been transferred into MSCs by viral and non-viral vectors and expressed at tumor sites for therapy **(C)**

 In this regard, research teams have investigated the potential of MSCs as a delivery system for widely recognized chemotherapeutic drugs, such as paclitaxel (PTX), doxorubicin (DOX), sorafenib, and gemcitabine. After loading with drugs MSCs can locally release their consignment through passive diffusion in the tumor stroma thereby leading to the death of cancer cells.^64^ The previous studies showed the effectiveness of PTX-loaded BM-MSCs and DP-MSCs in inhibiting the growth of certain malignant conditions including glioblastoma,^65^ and breast cancer^66^ through mitigation of the cell proliferation and inducing apoptosis. Moreover, conditioned medium obtained from ASCs-PTX significantly inhibited the proliferation of ovarian cancer cells compared with free PTX, and diminished PTX resistance in cancer cells.^67^ Likewise, the assessment of the potential antitumor activity of human DOX-loaded BM-MSCs in xenograft mouse models of thyroid or breast cancer demonstrated significant cytotoxic effects on tumor cells.^68^ Additionally, ASCs loaded with DOX showed impressive antitumor effects in l- B16F10 melanoma lung metastasis in vivo.^69^ A study has shown that human MSCs isolated from gingival papilla can serve as a reliable delivery system for gemcitabine release in an active form and in appropriate quantities to inhibit the proliferation of oral squamous cells.^70^ Another experimental study indicated that gemcitabine-loaded human BM-MSCs could dramatically repress the growth of human pancreatic malignant cells (Figure 4A).^71^

###  MSCs as a protective tool for the delivery of oncolytic viruses

 Oncolytic viruses, as anti-tumor biological compounds, are considered an innovative and promising therapeutic strategy for the amelioration of malignancies, which can selectively kill infected cancer cells by apoptosis induction.^72^ Oncolytic viruses with a natural affinity for tumor cells, can selectively target the malignant cells and lead to their lysis.^73^ Talimogene laherparepvec (T-VEC) is a genetically modified form of human herpes simplex virus type 1 (HSV-1). In 2015, T-VEC was approved by the United States Food and Drug Administration (US-FDA) as the first OV for melanoma treatment.^74^ In a late study, Zhang and colleagues reported that oncolytic HSV-1 can influence TME by decreasing the proportion of anti-inflammatory macrophages and elevating the presence of tumor-infiltrating lymphocytes. Furthermore, they represented that the combination of oncolytic HSV-1 and immune checkpoint modulators significantly extended the lifespan of the pancreatic tumor-bearing mice.^75^ However numerous factors contribute to the effectiveness of the virus in spreading within a cancerous tissue, including the quick elimination of the virus by the immune system and viral captivation by tissues and organs.^76,77^ Several research studies have indicated that MSCs can serve as eligible vehicles to protect OVs from neutralizing host effects, facilitate the targeted delivery of OVs, and improve their capacity to infect and eliminate cancer cells.^78^ The experimental findings demonstrated that MSCs loaded with oncolytic adenovirus promote virus replication leading to increased production of virus particles and a high accumulation of virions in tumors. Ultimately MSCs infected with oncolytic adenoviruses were able to effectively destroy hepatocellular carcinoma cells in vitro.^79^ Additionally, in vivo findings displayed that MSCs can prevent immune response by suppressing the release of interferon-γ (IFN-γ) from activated T cells. Also, MSCs enhanced the distribution and persistence of adenovirus in comparison to the injection of the virus alone in vivo.^80^ For enveloped OVs, MSCs can transport these viruses to tumor tissues through a process that involves hetero-cellular fusion. Ong et al introduced the oncolytic measles virus into bone marrow-derived MSCs and carried out in vitro co-culture experiments with human hepatocellular carcinoma cells.^72^ The findings indicated that the number of syncytia (cell fusions) increases when MSCs carry the measles virus, whereas this effect is not observed with non-enveloped viruses. Moreover, when high-titer anti-measles virus antibodies are present, virus-infected MSCs notably induce the formation of heterocellular structures when compared to the naked virus. These results align with the observations reported by of Castleton et al, who studied the use of MSCs to deliver the measles virus in a model of acute lymphoblastic leukemia. They suggested that using MSCs for OVs delivery could substantially extend survival and improve the effectiveness of anti-tumor interventions compared to using the virus alone.^81^ The different investigations demonstrated that MSCs with their unique abilities such as tumor tropism and immunosuppression help the virus to precisely reach the tumor site and enhance the virus persistence.^82,83^ In recent years, an increasing amount of evidence from both preclinical and clinical studies has highlighted the immunosuppressive abilities of MSCs, as they can inhibit the activity of specific immune cell types, such as T and B lymphocytes, as well as NK cells. Consequently, this modulation extends to affecting the function of monocytes, DCs, and macrophages.^84-87^ MSCs influence the activation, growth, maturation, cytokine release, and cytotoxic capabilities of both innate and adaptive immune cells.^88^ Certainly, MSCs can decrease cytokine production by helper T cells, diminish the cytotoxic effects of effector T lymphocytes,^89^ impede the differentiation of B lymphocytes, and hinder their capacity to release immunoglobulins.^90,91^ Additionally, they can confine INF-γ secretion by NK cells and attenuate their cytotoxic potential. Furthermore, MSCs hinder the differentiation of CD14 + monocytes and CD34 + progenitor cells into fully mature DCs.^92^ Crucially, MSCs foster the development of regulatory immune subgroups, such as CD8 + CD28− T lymphocytes,^93^ CD4 + CD25 + FOXP3 + T lymphocytes,^94^ IL-10 producing B lymphocytes and DCs (Figure 4B).^95,96^ Hence, the inhibition of immune cell activities and the promotion of regulatory immune cell subsets may play beneficial roles in enhancing immunosuppressive capabilities of MSCs. These functions are essential MSCs features in protecting OVs from immune system clearance guaranteeing enhanced OV spread and increased viral persistence.^80^ It has been acknowledged that MSCs-mediated delivery of OVs in animal models of solid tumors has shown successful outcomes in improving hepatocellular carcinoma,^97^ glioblastoma,^98^ glioma,^99^ colorectal cancer,^100^ prostate cancer,^100^ and lung metastases of breast carcinoma.^101^

###  MSCs as a new platform for the delivery of suicide genes

 Another modality for cancer treatment is using suicide genes which provide the possibility for selective destruction of malignant cells without harming the surrounding normal cells.^102^ Suicide gene therapy or gene-directed enzyme prodrug therapy, is based on the transfer of a foreign gene that encodes an enzyme into cancer cells. This enzyme converts a prodrug into toxic metabolites leading to the death of the cancer cells.^103^ The bystander effect is an intriguing characteristic of the suicide gene resulting in the elimination of both cancerous cells in which the toxic metabolites are formed and the adjacent non-transgenic cancer cells.^104^ The major limitation that restricts the success of suicide gene cancer therapy is the low efficiency in delivering and expressing the therapeutic genes.^105^ To address the challenges associated with using suicide gene therapy for treating tumors, scientists have identified the beneficial role of MSCs due to their homing ability to target cancerous cells, as the appropriate cellular carriers for suicide genes.^106^ Expression of the suicide genes by the MSCs at the tumor local converts the administered non-toxic prodrug to an active toxic compound that is fatal to tumor cells.^107^ The herpes simplex virus thymidine kinase complexed with ganciclovir (HSV-TK/GCV system) and yeast cytosine deaminase (CD) with 5-fluorocytosine (5-FC) are the most common enzyme-prodrug complexes that in combination with MSCs can target different types of tumors. ^108^ For example, the injection of hMSCs transfected with the suicide gene CD, which was followed by the administration of 5-FU in a mouse model with gastric cancer was found to suppress the growth of the tumor.^109^ Furthermore, in vitro and in vivo experiments demonstrated that MSCs expressing cytosine deaminase::uracil phosphoribosyl transferase (CD::UPRT) can trigger complete tumor regression in prostate cancer models.^110^ CD::UPRT can convert the non-toxic 5-fluorocytosine into the cytotoxic anti-tumor drug known as 5-fluorouracil.^111,112^ Recent findings showed the safety of adipose tissue–derived allogeneic MSCs which carry herpes simplex virus-thymidine kinase (HSV-TK) gene, as a suicide gene therapy in patients with recurrent glioblastoma.^113^ Another investigation displayed that when MSCs expressing the HSV-TK were used in conjunction with the ganciclovir (GCV) prodrug, it exhibited its possible anti-tumor effectiveness both in laboratory settings and in mice models using the human glioblastoma cell line U87TK. During this, MSCs preserved cell proliferation, karyotype stability, and retained their MSCs characteristics. Moreover, genetic modification had a notable impact on their secretory profile, leading to a substantial increase in various anti-tumor immune soluble factors such as IFN-γ, IL-2, MCP-1, and IL12p40 (Figure 4C).^114^ Several studies in the recent decade have shown that anticancer drug-conjugated nanoparticle-loaded MSCs, due to the increasing migration activity of MSCs and controlled and gradual drug- release in target tissue, can be introduced as a novel tool in cancer therapy.^115-117^

###  MSCs-derived exosomes for cancer therapy 

**Figure 5:**
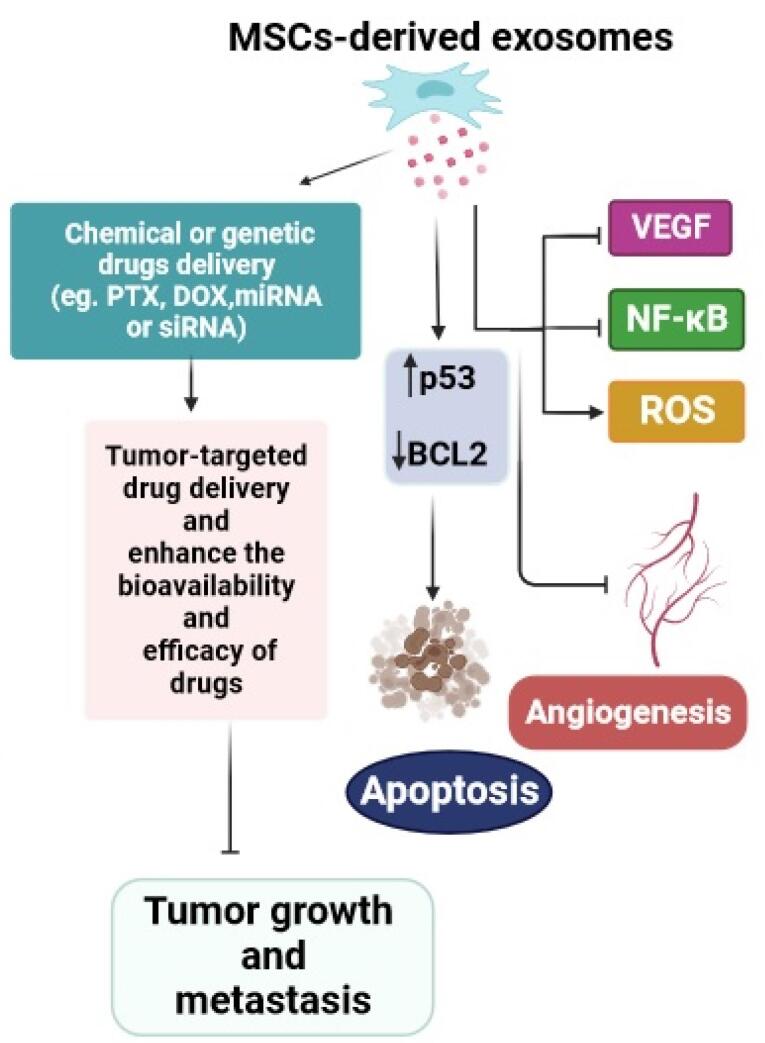
Mesenchymal stem cells-derived exosomes as an attractive cell-free approach in cancer therapy. The web-based application BioRender was employed to design the figure. MSCs-derived exosomes as an attractive cell-free approach in cancer therapy. MSCs-exosomes can be loaded with various types of compounds such as chemical or genetic drugs to target tumor tissues and inhibit tumor growth and metastasis. Exosomes can also influence cancer cells by promoting apoptosis and preventing angiogenesis

 Extracellular vesicles (EVs) are a diverse group of small, membrane-enclosed structures ranging from approximately 30 to 1000 nanometers in diameter. These EVs are actively released by all types of cells into the extracellular space and plentifully found in different body biofluids such as saliva, synovial fluid, amniotic fluid, ejaculate, cerebral spinal fluid, milk, and even urine.^118-121^ These spherical, bilayered particles are rich in proteins, lipids, nucleic acids, and other bioactive metabolites.^122,123^ EVs, as a modern messaging system, can mediate cell-to-cell contact and intercellular crosstalk transfer via transferring their bioactive cargo to recipient cells.^124^ Different types of EVs can be broadly categorized into three major groups based on their mode of biogenesis: exosomes, ectosomes, and apoptotic bodies.^125^ Exosomes generally constitute the smallest EVs, less than 150 nm in size. They are produced as intraluminal vesicles in the endosomal system through the fusion of multivesicular bodies with the plasma membrane.^126-128^ In contrast, ectosomes, or microvesicles, are larger, varying from approximately 100–1000 nm, and are secreted by direct outward budding and shedding from the plasma membrane.^129^ Similarly, exosomes and ectosomes play crucial roles in intercellular communications.^130^ Tetraspanins form a diverse superfamily of small transmembrane proteins that are present in both types of EV and are accepted as a critical cellular effector during the biogenesis of these EVs.^131,132^ Apoptotic bodies are a peculiar type of EV with a large size (1000–5000 nm) secreted by cells that have undergone apoptosis, or programmed cell death.^133^ MSCs from different tissue sources possess the ability to generate and release various types of EVs.^134^ Exosomes are the most important secreted extracellular particles from MSCs with a diameter of 30–100 nm that not only express common surface biomarkers such as CD81 and CD9, but also express MSCs surface markers, such as CD29, CD44, CD73, and CD90.^135,136^ MSCs-derived exosomes owing to containing multiple therapeutic cargoes including proteins, lipids, nucleic acids (DNAs and RNAs), and metabolites, have distinct effects on cell interactions through various mechanisms.^137^ Furthermore, MSCs-derived exosomes have been proposed as a prospective and powerful cell-free-based tool for combatting cancer due to having numerous exclusive features such as low immunogenicity, biosafety, biocompatibility, prolonged circulation time, sustained release, and tumor-particular homing.^138-140^ Angiogenesis, as a process involved in new vessel formation, can accelerate tumor growth, while exosomes derived from MSCs can prevent angiogenesis by regulating VEGF expression. The study by Miranda and colleagues found that exosomes derived from MSCs can inhibit angiogenesis in prostate cancer (PC3) cells. This inhibition is achieved by reducing the secretion of the pro-angiogenic factor VEGF, suppressing the activity of the transcription factor NF-κB, and promoting the production of reactive oxygen species within the cancer cells.^141^ The researchers further investigated the anticancer features of exosomes by examining how MSCs-derived exosomes affect the expression of genes involved in angiogenesis and apoptosis in several cancer cell lines. They indicated that MSCs-released exosomes can induce apoptosis by enhancing p53 gene expression and decreasing BCL2 gene expression, meanwhile impeding the proliferation of cancer cells.^142^ The available evidence suggests that MSCs exosomes can serve as effective nano-carriers for the delivery of antitumor drugs/ genes (miRNA or siRNA), facilitate tumor-targeted drug delivery, and enhance the bioavailability and efficacy of drugs.^143^ Ono et al found that MSCs-derived exosomes can suppress the cell cycle and promote dormancy in breast cancer cells via secretion of miR-23b, leading to the inhibition of migration and metastases of breast cancer cells.^144^ Besides, it has been presented that MSCs-derived exosomes containing miR-379 can inhibit growth of T47D breast cell lines expressing miR-379, indicating their potential as an effective cell-based therapy for targeted therapy of breast cancer.^145^ Small interfering ribonucleic acid (siRNA), which selectively inhibits a target gene, possesses great features in cancer treatment. Recently, a group of researchers found that MSCs-released exosomes carrying polo-like kinase 1 (PLK-1) siRNA leading to apoptosis and necrosis in bladder cancer cells.^146^ Pascucci et al were the first to investigate the ability of MSCs-derived exosomes to encapsulate and deliver PTX as a chemotherapeutic agent. The obtained data from this study showed that exosomes have good efficacy to uptake/release PTX, indicating that MSCs-derived exosomes can be a new method for drug delivery.^147^ The results of another investigation confirmed the anticancer function of exosomes loaded with PXT by reducing the tumor size and inhibiting the distant metastasis of breast cancer cells in the liver, spleen, and kidneys.^148^ Further, it has been reported that the uptake and cytotoxicity of MSCs-derived exosomes loaded with DOX are significantly higher than the free DOX in the osteosarcoma MG63 cell line. Therefore combined exosome-DOX was introduced as a super candidate for osteosarcoma treatment.^149^ Collectively, MSCs-derived exosomes exhibit distinct characteristics such as paracrine effects, immunomodulatory capabilities, gene transfer potential, biocompatibility, and stability. These attributes make them valuable biological tools for enhancing the efficacy and safety of conventional anticancer therapies (Figure 5).^150,151^

## Signaling pathways regulated by MSCs

 Numerous studies in recent decades have suggested that different signaling pathways are implicated in the development and progression of cancer.^152^ Based on different evidence, MSCs show a high capability to inhibit cancer through the modulation of diverse signaling pathways in the TME.^153^ In this section, several primary cancer-related signaling pathways influenced by MSCs were chosen for a thorough assessment of their anti-tumor effects. The Wnt signaling pathway, as one of the most important pathways in controlling the processes of cell growth and specialization, plays a prominent role in cancer progression.^154^ MSCs can suppress the growth of cancer cells by overexpressing P21CIP1 and P27KIP1, which in turn inhibit the Wnt signaling pathway by down-regulating c-Myc and cyclinD2 and promoting the production of the tumor suppressor Dickkopf-related protein 1.^155^ The wnt/β-catenin pathway promotes tumorigenesis in various types of cancer.^156^ The research conducted by Visweswaran et al has proven that factors derived from ASCs can inhibit cancer cell growth by reducing the expression of activated protein β-catenin and cyclin D1, and key target proteins of the Wnt pathway, and also can induce apoptosis via inhibition of the anti-apoptotic protein expression such as Bcl-XL.^157^ Blocking the β-catenin signaling pathway had a profound impact on preventing both tumor formation and metastasis in breast cancer cells overexpressing HER2.^158^ The PI3K/AKT/mTOR signaling pathway, as one of the most common intracellular pathways, can influence various downstream target proteins and is implicated in tumorigenesis, proliferation, drug resistance, the emergence of stem-cell-like traits, invasion, and metastasis of malignant cells.^159,160^ MSCs can induce cell cycle arrest and reduce cancer growth by inhibiting proliferation-related signaling pathways, such as the phosphatidylinositol 3-kinase/protein kinase B (PI3K/AKT).^161^ Inhibition of AKT was showed in a Kaposi’s sarcoma model in which intravenously injected MSCs migrated to tumors and significantly suppressed tumor growth. The JAK/STAT signaling pathway is a key factor in the progression of cancer, serving as a driver of cancer growth and metastasis within tumors, or as a regulator of immune surveillance.^162^ Thus, suppressing the JAK/STAT pathway is encouraging for remedying various illnesses. He et al have presented that the MSCs-conditioned medium impedes the STAT3 signaling pathway in breast cancer cells and inhibits tumor progression. This finding indicates that paracrine-soluble factors secreted by MSCs could regulate JAK/STAT signaling and suppress the growth of breast tumors.^163^

## Limitations and disadvantages of MSCs-and exosome-based therapies for cancer

 During the past decades, there has been a discernible advancement in MSCs-based therapies for different cancer type.^164^ In spite of the amazing therapeutic potential of MSCs, there have been some inconsistent results from the use of MSCs in preclinical and clinical studies that may be caused by the heterogeneity of them.^165^ The heterogeneity of MSCs depends on different factors, including cell origin (tissue), the conditions of donors (age, diseases, or unknown factors), dosage, administration route, expansion protocol, and culture passage number of cells.^166,167^ Therefore, strategies and methods are needed that can manage these challenging issues. The use of standardized procedures for MSCs isolation, characterization, and expansion is critical to mitigating variability and improving the clinical efficacy of MSCs.^168^ Also, the dosage, route, and timing of administration should be optimized.^169^ Although the ideal MSCs dosage is still unknown, systematic intravenous injection (IV) of MSCs at a dose of 100–150 million cells per patient has been recommended to be beneficial for cancer therapy approaches.^170^ Meanwhile, many studies have shown that IV infusion of MSCs leads to the entrapment of cells in the lung, resulting in a reduction in the population of cells and the homing of less than 1% of them to target sites.^171,172^ Another of the most important reasons for MSCs utilization in the therapeutic area is their differentiation potential and immunomodulatory potency.^173^ These properties are affected by the specific tissue source from which the MSCs are derived, the age and health status of the donor, and the culture conditions and environment in which the MSCs were grown and expanded outside the body before being administered.^174^ So, MSCs derived from different tissues have some divergence in their proliferative and differentiation capacities and levels of secreted immunoregulatory cytokines. Furthermore, multiple studies provide evidence that aging causes a considerable reduction in the differentiation ability and immunomodulatory function of hMSCs.^175-177^ Among the major challenges to the application of MSCs to treat diverse pathologies is the need for large and sufficient amounts of cells that can only be obtained through long-term ex vivo expansion.^178^ Genomic instability and chromosomal aberrations are recognized as the most important occurrences during long-term culture that elevate the risk of tumorigenicity of MSCs after transplantation in patients. Therefore, MSCs can be unsafe for clinical use.^179,180^ In this regard, a number of studies have suggested exosomes derived from MSCs as an appropriate substitute option for overcoming the restrictions and disadvantages associated with cell-based therapy.^181,182^ Exosomes, the natural nanocarriers of bioactive signals, due to their hydrophilicity and small size, can even cross the blood-brain barrier and placental barrier, exerting favorable therapeutic effects in different types of disease.^183,184^ So far, several clinical trials have confirmed the helpful effects of MSCs-exosomes on the improvement of patients with cancer.^185^ Nevertheless, employing exosomes in clinical trials encounters challenges and limitations. The outstanding obstacles include the absence of a standardized exosome extraction and purification procedure, weak characterization, low yield of exosomes, sterility and biosafety, long-term maintenance, optimal therapeutic dosage, injection root,, and a short half-life.^186,187^ Exosome isolation is a determining process for getting a pure and uncontaminated sample with a high concentration, which facilitates precise evaluations of the functions and characteristics unique to exosomes.^188^ The heterogenicity of exosomes, arising from differences in their size, contents, and surface markers, poses a significant problem for efficient isolation, purification, and characterization of them.^189^ In order to overcome the heterogeneity of exosomes, it is necessary to recruit an efficient separation strategy, enabling the distinction of exosomes from various sample matrices.^190^ Currently, several techniques have been established for the sorting of exosomes based on their density, size, and surface proteins, including ultracentrifugation, size-exclusion chromatography, immunoaffinity, and polymer-based precipitation. These procedures, along with analysis methods such as nanoparticle tracking, electron microscopy, flow cytometry, and western blots, have helped advance the use of purified exosomes as an efficient drug delivery vehicle for cancer therapy.^188,190-192^ Nevertheless, the approaches used to extract, purify, and store exosomes need to conform to Good Manufacturing Practice (GMP) standards in order to generate a product with high biosafety to enter clinical settings.^193^ Determining the optimal dose, as one of the impressionable factors in exosome-based therapy, is affected by some considerations, such as the administration route and half-life.^194^ Due to the short circulation half-lives of exosomes and rapid fluid turnover (blood, sweat, or tears), systemically and locally administered injections cause rapid clearance from blood circulation and cumulation of the exosomes in the spleen, liver, and lung.^195,196^ Thus, the short half-life is an important limitation for the effective transfer of exosomes to damaged tissue and the continuity of their presence in the target location.^197^ Despite considerable advances in the study of MSCs and their derived exosomes, there are numerous issues and restrictions that have hindered their clinical use and should be addressed more in future research.

## Conclusion

 Cancer is one of the most significant causes of people life-threatening worldwide. Even though the disease conditions are highly progressive, there is no definitive cure, and nearly all current therapeutic approaches aim to control the advancement and progression of cancer. MSCs-associated cell therapies are considered promising treatment candidates with potential ameliorating effects on disease progression. MSCs due to having robust tumor-tropic capacity can migrate to tumor tissues, therefore these cells are a good option for targeted delivery of different chemical and genetic agents to tumor sites, reducing the side effects of various drugs on healthy tissues. They also can modulate inflammation conditions in the TME via producing higher levels of paracrine factors and suppression of T cell proliferation, NK cells activation, and DCs maturation, as a result, can be the favorite therapy for controlling cancer. In addition, MSCs-derived exosomes, as a cell-free tool, offer unique advantages for use in cancer therapy and are notable for the delivery of several therapeutic molecules including chemotherapeutic drugs, miRNAs, specific siRNAs, and suicide gene mRNAs. Overall, the numerous experimental studies and clinical trials provide promising results regarding the use of MSCs in cancer therapy and confirm the potential of MSCs to combat cancer. In conclusion, hopeful progressions have been made in oncology research, so MSCs-based therapies can be a surprising revolution in medicine and the treatment of patients suffering from cancer.

## Acknowledgments

 We acknowledge the Hematology and Oncology Research Center at Tabriz University of Medical Sciences, Iran for their support.

## Competing Interests

 The authors declare that there are no conflicts of interest.

## Consent for publication

 The authors consent for the publication of the manuscript.

## Ethical Approval

 Not applicable.
